# Cell Toxicity and Autophagy in A549 Cells Treated With Surface‐Functionalized Graphene Derivatives

**DOI:** 10.1002/jat.4869

**Published:** 2025-09-09

**Authors:** Tae Yun Park, Soo Young Kim, Chang Seok Park, Won Young Kim, Jae‐Woo Jung, Jae‐Yeol Kim, Jong Wook Shin

**Affiliations:** ^1^ Division of Respiratory and Critical Care, Department of Internal Medicine Seoul Metropolitan Government‐Seoul National University Boramae Medical Center Seoul South Korea; ^2^ Department of Materials Science and Engineering Korea University Seoul South Korea; ^3^ Department of Internal Medicine Chung‐Ang University College of Medicine Seoul South Korea

**Keywords:** autophagy, cell toxicity, graphene derivatives

## Abstract

Graphene oxide and its derivatives have unique physical and chemical properties with applications in many different fields. However, their biological effects and mechanisms of intracellular toxicity have not been completely clarified. In this study, we investigated the cytotoxic and autophagic activities of graphene oxide and its derivatives in A549 human lung carcinoma cells. In the experimental procedure, A549 cells were treated with graphene oxide (GO), dodecylamine‐oxidized graphene (DA‐GO), reduced graphene oxide (rGO), and sodium dodecyl sulfate‐reduced graphene oxide (SDS‐rGO), and their cytotoxicity and protein expression levels were measured. Treating A549 cells with each type of graphene induced a concentration‐dependent toxic effect on the cells, with no obvious cytotoxicity at low concentrations (32 𝜇g/mL). However, those treated with graphene with dodecylamine and sodium dodecyl sulfate functional groups exhibited high toxicity compared to its native form at high concentrations (> 100 𝜇g/mL). Cells exposed to the graphene materials exhibited increased conversion of LC3A/B‐I to LC3A/B‐II depending on concentration, indicating increased autophagy activity. They also exhibited reduced levels of mTOR protein, a negative regulator of autophagy, compared to a control group for all graphene materials. However, concentrations of beclin‐1, a positive regulator of autophagy, were lower for all types of GO. These findings suggest that graphene exposure may induce beclin‐1‐independent autophagy in a noncanonical manner. We hypothesize that this may be a result of the involvement of apoptosis‐associated substances that suppress autophagy. However, the exact mechanisms of the autophagy process are not well understood, and further research remains necessary.

## Abstract 2

This study investigates cytotoxicity and autophagy in A549 cells treated with various graphene derivatives. Functionalized graphene induced concentration‐dependent toxicity and activated autophagy through a Beclin‐1‐independent pathway. Findings suggest that graphene may trigger noncanonical autophagy via apoptotic signaling, highlighting implications for biomedical safety and nanomaterial application.

## 1 Introduction

Graphene‐based nanomaterials have drawn great attention over the last decade. Because of its unique physicochemical properties, graphene and its derivatives have shown great potential in many biological and medical applications such as gene/drug delivery, imaging, cellular probing, cellular differentiation, and photothermal therapy (Chen et al. 2012; Goenka et al. 2014; Hu et al. 2012; Yang et al. 2012; Zhang et al. 2010). Despite the extensive applications of graphene, graphene nanomaterials might have side effects on health. Previous studies demonstrated that the graphene materials can induce cytotoxic effects in many other cell lines (Lalwani et al. 2016; Lewinski et al. 2008; Singh 2016; Zhang et al. 2016). Duch et al. (2011) reported that graphene oxide (GO) induces the production of reactive oxygen species (ROS) in alveolar macrophages and epithelial cells, leading to inflammation and culminating in cell apoptosis due to impaired mitochondrial respiration. In another study, graphene exhibits toxic effects on human fibroblast cells, resulting in reduced cell adhesion and the facilitation of cell apoptosis (Wang et al. 2011). In addition to the lung cells and fibroblasts described above, cytotoxic effects have also been observed in hepatocytes and intestinal cells (Ahamed et al. 2020b; Cebadero‐Domínguez et al. 2022). These cytotoxic effects are various depending on physicochemical characteristics (size, morphology and functional groups) and concentration (Ou et al. 2016; Rhazouani et al. 2021).

Different forms of graphene and its derivatives may reveal different physical/chemical properties and biological toxicities. The toxic effect of graphene material may be attributed to many different mechanisms, including ROS production, DNA damage, apoptosis, autophagy, and those mechanisms are still under study (Ou et al. 2017). Among them, research on the autophagy of graphene exposure is increasing in recent years. Autophagy is the biological process that includes the enzymatic degradation of a cell's components (such as damaged organelles or proteins) through the lysosomal pathway. Active autophagy generally leads to increased phosphorylation of Beclin‐1 and conversion of LC3B‐II and p62 expression levels. This process maintains cellular homeostasis as a recycling process, but excessive autophagic response is closely associated with the induction of autophagic cell death.

There are some studies on autophagic activities of graphene, but the underlying process is not clearly identified and it has not been extensively investigated.

Hence, the purpose of this study is to investigate the effects of graphene derivatives on cell viability, autophagy activity, and autophagy regulating molecules in human lung carcinoma A549 cells. The findings may help explain the toxicity and mechanisms of cells after different GO derivatives exposure, and it can provide insight into the safe application of GOs in humans.

## 2 Materials and Methods

### 2.1 Synthesis of GO

GO was manufactured through a modified Hummers and Offeman (1958) method. Graphite powder (2 g of 99.9995%, Alfa Aesar, 200 mesh) was mixed with NaNO_3_ (2 g) and concentrated H_2_SO_4_ (100 mL) for 24 h in an ice bath. Next, KMnO_4_ (12 mg) was added to the mixture gradually. After mixing the solution, the ice water bath was eliminated, and the solution was stirred at a temperature of 35°C until it turned into a highly viscous fluid. Afterward, 100 mL of pure water and H_2_O_2_ were sequentially added to the viscous suspension, and the mixture was centrifuged at 6000 rpm and rinsed with HCl and water. The centrifuging and rinsing processes were repeated at least five times. Lastly, the GO was dehydrated at 50°C for 1 day by using a vacuum oven.

### 2.2 Synthesis of Dodecylamine‐Modified GO (DA‐GO)

GO was scattered in 200 mL of deionized water (2 mg/mL). Ultrasonication was conducted on scattered GO for 30 min through a WUC‐A03H (DAIHAN Scientific, Korea) sonicator. Centrifugation was then used to eliminate the unexfoliated GO for 15 min at 3000 rpm. Afterward, 100 mL of the coffee‐colored dispersion was moved to a beaker. The mixture of dodecylamine (DA, 0.1853 g) and ethanol (100 mL) was added to the GO dispersion, and the final mixture was gently mixed for 2 h at room temperature. Nucleophilic substitution developed between the amine functional group of DA and the epoxy functional group of the GO. The DA‐functionalized GO suspension was cleansed with ethanol to eliminate excessive DA that was attached to the surface of the modified GO. Then, it was treated with deionized water to eliminate the extra ethanol. The dark brown powder was dehydrated by using a vacuum at 60°C for 1 day to generate the final product, dodecylamine‐modified graphene oxide (DA‐GO).

### 2.3 Synthesis of Reduced Graphene Oxide (rGO)

rGO was synthesized from GO using hydrazine hydrate (Gómez‐Navarro et al. 2007). First, GO (0.1 g) was scattered in deionized water (100 mL) through ultrasonication. Next, hydrazine hydrate (1 mL, Sigma Aldrich, reagent grade N2H4 50%–60%) was mixed in the GO dispersion.

After stirring the mixture for 10 min, it was moved to an oil bath equipped with a water‐cooled condenser and heated at 90°C for 1 day. The reaction product was filtered and cleansed with deionized water to remove excess hydrazine hydrate. The final product was dehydrated in a vacuum oven at a temperature of 60°C.

### 2.4 Synthesis of Sodium Dodecyl Sulfate‐Modified Reduced Graphene Oxide (SDS‐rGO)

For sodium dodecyl sulfate (SDS) modification (Hsieh et al. 2013), rGO was mixed in deionized water (200 mL) with a concentration of 2 mg/mL by using ultrasonication for 30 min using a VCX‐750 probe type sonicator (Sonics & Materials Inc., USA). After 14.419‐g SDS (Sig ma‐Aldrich, ACS reagent, ≥ 990%) was added to the rGO dispersion, the dispersed solution was stirred for 2 h at room temperature. The SDS‐rGO dispersion was washed with deionized water to remove the extra SDS. Finally, the black powder was dried through vacuum at 60°C for 1 day.

### 2.5 Cell Culturing and Treatment With GO Derivatives

Human lung carcinoma A549 cell lines were obtained from ATCC and cultured in Dulbecco's modified Eagle's medium (Gibco), supplemented with 10% fetal bovine serum (FBS, Gibco) and antibiotics (streptomycin/penicillin, Gibco), at a temperature of 37°C in a 5% CO2 atmosphere. We used four types of GO derivatives in our experiments: GO, DA‐GO, rGO, and SDS‐rGO. For each treatment, the A549 cells were seeded in appropriate vessels (96‐well plate, 100 pi dishes and 10‐mm diameter) with proper seeding numbers for each experiment and cultured overnight. Then, the cultured cell medium was treated with each type of GO at each planned concentration for 24 or 48 h.

### 2.6 Cytotoxicity Assay

The MTT [3‐(4, 5‐dimethylthiazol‐2‐yl)‐2, 5‐diphenyltetrazolium bromide] assay was performed to evaluate the cytotoxicity of each type of GO. Briefly, MTT solution (5 mg/mL) was added to Gibco phosphate‐buffered saline. Then, sterilization of the solution was performed by a syringe filter (0.2‐μm pore size, GVS). A549 cells were planted at a density of 1 × 10^4^ cells per well in the 96‐well plate and incubated for 24 h. The cells were separately treated with graphene derivatives (GO, DA‐GO, rGO, and SDS‐rGO) from 400 to 3.125 μg/mL, which were acquired by the half‐dilution method into the culture medium. Cultured cells in the medium without GO derivatives were used as the control. The blank consisted of media without cells or GO. The cells were washed two times with sterile cold PBS after 24 and 48‐h incubation. Next, 10% MTT solution in medium was applied to each well and incubated additionally for 4 h at 37°C. Afterward, 10% SDS solution (100 L) was applied to each well and incubated again for 4 h at 37°C to dissolve formazan residue completely from viable cells. The liquefied samples were stirred for 30 s until they were thoroughly mixed. Next, the samples underwent centrifugation at 14,000 rpm for 30 min. The optical density was evaluated using a microplate reader (SpectraMax 340PC384, Molecular devices) at 540 nm. A reference wavelength was defined as 670 nm. The experiments were repeated three times.

### 2.7 Protein Extraction and Western Blot Analysis

The GO type was classified into three categories (low, medium, and high) according to its concentration. The concentrations include 5, 50, 200 𝜇g/mL for each GO type. Total proteins were extracted from graphene‐treated cells, which were rinsed twice with cold PBS and lysed in the PRO‐PREP solution. The extracted elements were incubated for 20 min, and the next, it were centrifuged at 15000 rpm at 4°C for 30 min. The supernatants containing total proteins were gathered, and protein concentrations were measured using the SMART BCA Protein Assay Kit. Proteins were extracted in equivalent amounts by conducting sodium dodecyl sulfate‐polyacrylamide gel electrophoresis (SDS‐PAGE) and then transferred onto polyvinylidene fluoride (PVDF) membranes. The membranes were treated with 5% nonfat milk for blocking and were incubated for 24 h at 4°C with gentle shaking. Afterward, the membranes were incubated with proper secondary antibodies, and protein bands were identified by using Dogen Pico or Femto Chemiluminescence Kit. Quantification of protein band densities was performed by using an imaging processing program (Image Lab). To evaluate the autophagy activity, the extent of LC3A/B conversion (LC3A/B‐I to LC3A/B‐II) was identified by performing western blot analysis. Conversion of LC3A/B was defined as the following formula: band density of LC3A/B‐II divided by band density of LC3A/B‐I. As an internal control, we used the expression of glyceraldehyde 3‐phosphate dehydrogenase (GAPDH).

## 3 Results

### 3.1 Fourier Transform Infrared (FTIR) Spectra of Four Types of GO Derivatives

**FIGURE 1:**
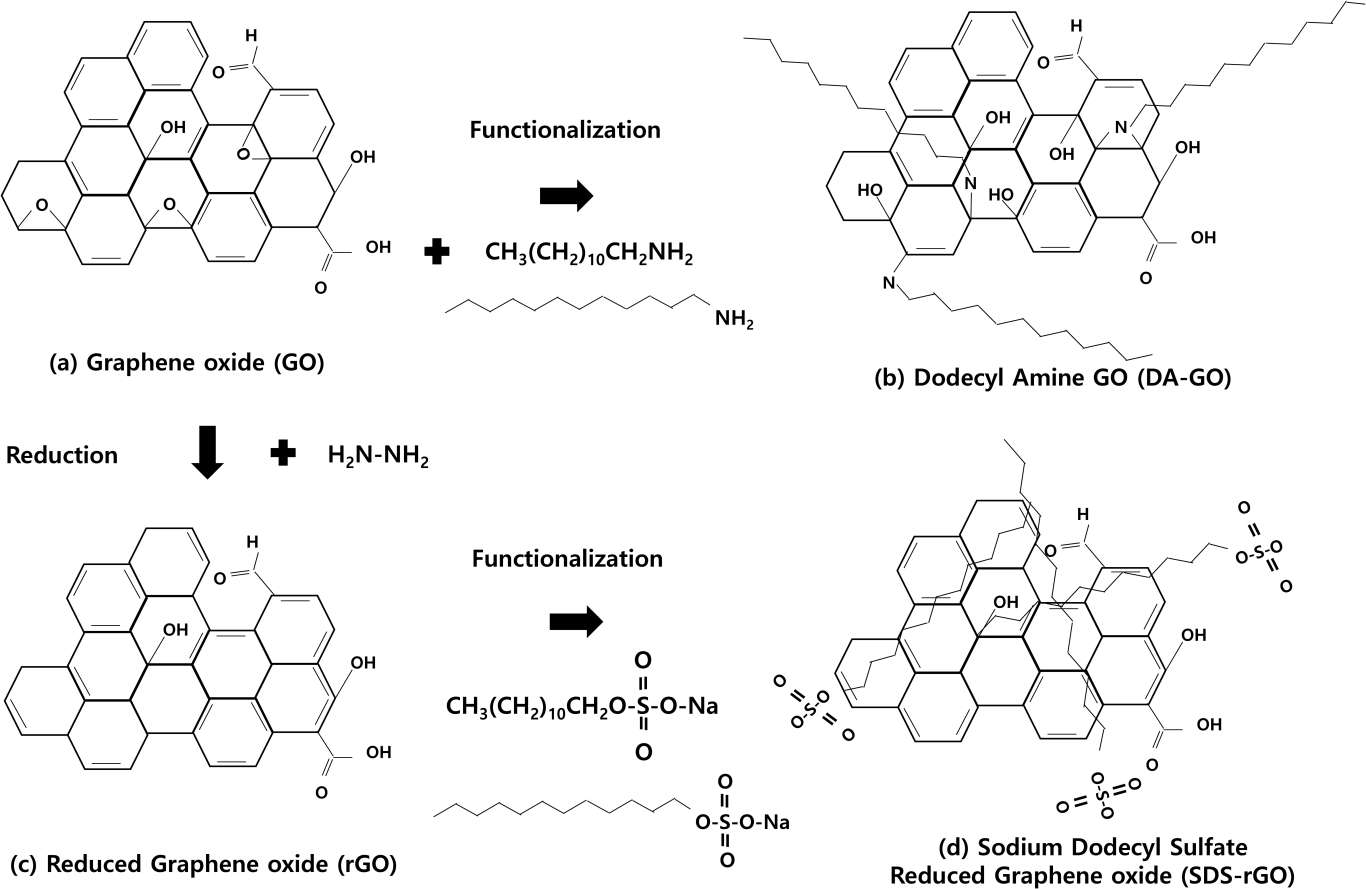
Structure of (a) GO, (b) rGO, (c) DA‐GO, and (d) SDS‐rGO.

**FIGURE 2:**
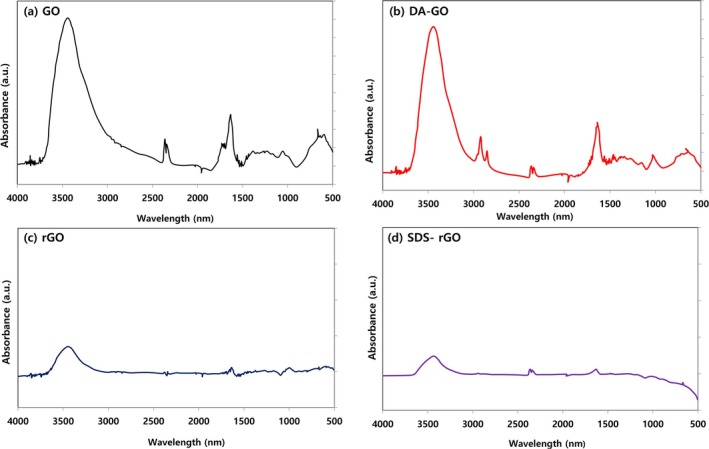
FTIR spectra of (a) GO, (b) DA‐GO, (c) rGO, and (d) SDS‐rGO.

The predicted chemical structure of each graphene sample was shown in Figure 1. In our previous work, the FTIR spectroscopy was performed to evaluate the structural analysis of graphene oxide (GO) derivatives (Park et al. 2015). The FTIR spectra result of (a) GO, (b) DA‐GO, (c) rGO, and (d) SDS‐rGO was shown in Figure 2. A broad absorption band at 3437 cm^−1^, corresponding to OH functional groups, was shown for all the samples. The observed widespread O‐H stretching bands indicate that strong hydrogen bonding networks are forming between sheets and between functional groups, beyond simple hydroxyl introduction. These hydrogen bonds not only demonstrate high functionalization efficiency but also improve dispersion properties by increasing colloidal stability and water adsorption and are important spectroscopic indicators that can predict the strength of cell membrane‐protein interactions. The rGO and SDS‐rGO still include some oxygen bonds, but their oxygen contents decreased compared to GO after reduction. Another absorption peak at 1636 cm^−1^ also appeared for all the samples, demonstrating the existence of the graphitic sp2 bond. The peak scales around from 1700 to 1733 cm^−1^ (C=O stretch of –COOH) and 1272 cm^−1^(epoxy C–O–C), representing the carboxyl and epoxy functional groups, respectively. However, these peaks were not found for other samples, implying that these functional groups interacted with DA in DA‐GO and underwent reduction in RGO and SDS‐rGO. The remaining absorption peaks in GO were shown at 1380 (CH^3^ bending) and 1056 cm^−1^, that typically appear in primary alcohol (Bissessur et al. 2006; Wang et al. 2010). The new peaks observed at 2955, 2922, and 2852 cm^−1^ in DA‐GO specimens suggest stretching vibrations of the C‐H group, consisting of CH^3^, CH^2^, and CH groups, respectively. Another peaks at 1644 (amide C=O), 1457 (N–H bending), 1265 (C–N stretch), and 1012 cm^−1^ (phenolic –OH) shown in DA‐GO represent stretching vibrations of the carbonyl group from an amide‐carbonyl bond, an amine group, an amine stretching, and a primary alcohol in phenolic compounds, respectively (Kuila et al. 2012; Wang et al. 2009). The presence of these peaks suggests that GO was successfully changed to DA‐GO. The absorption peaks at 998 and 990 cm^−1^ for the rGO and SDS‐rGO come from stretching vibrations of C‐H out‐of‐plane, and this implies adequate reduction of GO. A new absorption peak at 1168 cm^−1^ for SDS‐rGO arose from S=O stretching and suggested that small amounts of SDS remained on the surface of rGO (Pavia et al. 2015). These results suggest that all the samples were modified as intended. The FTIR peak summary for GO derivatives is summarized in Table S1.

### 3.2 The Cytotoxicity of A549 Cells Treated With GO Derivatives

**FIGURE 3:**
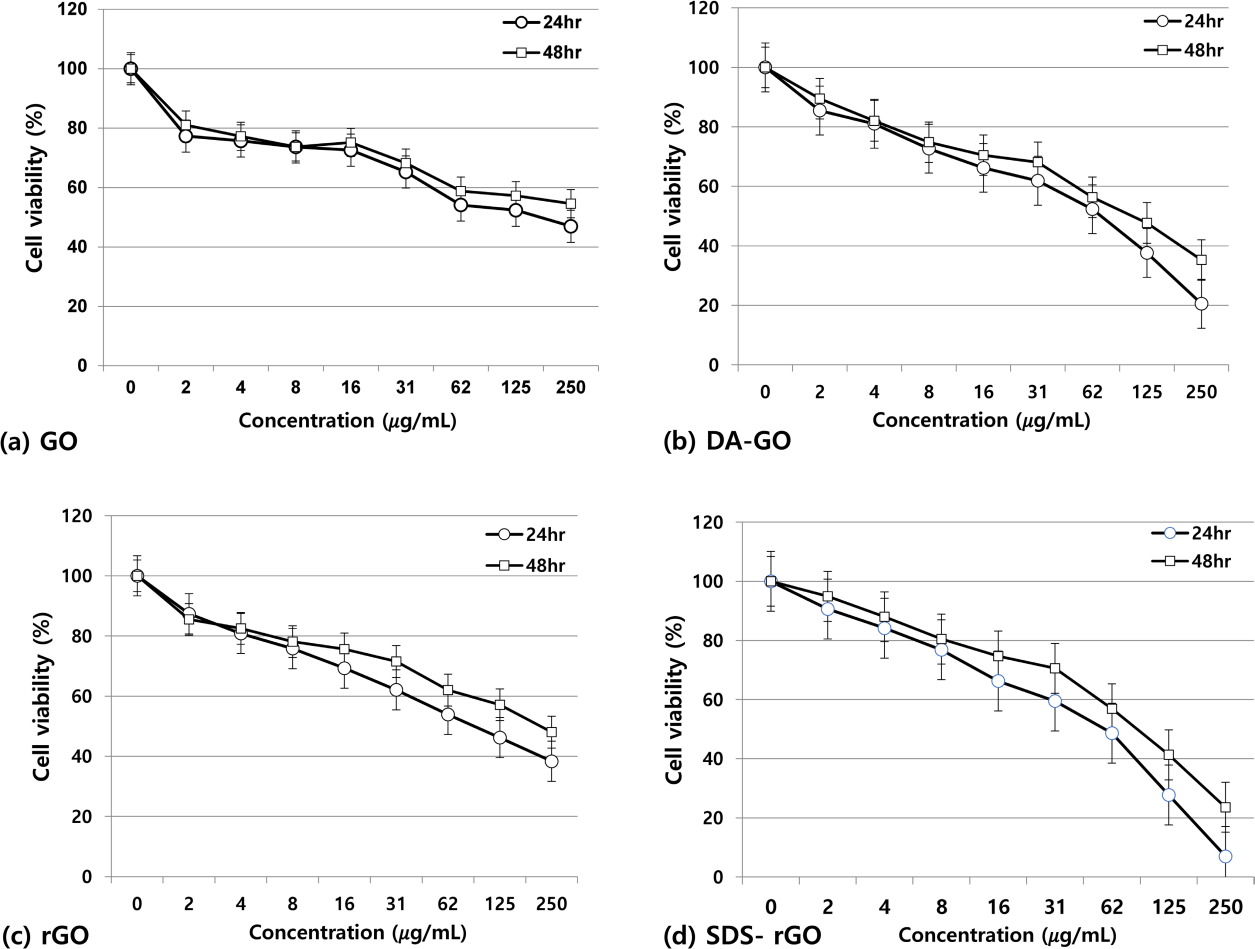
The MTT Assay of A549 cells exposed to (a) GO, (b) DA‐GO, (c) rGO, and (d) SDS‐rGO.

The cytotoxicity of GOs treated A549 cells was determined by performing the MTT assay (Figure 3). Cell viability was linearly decreased with increasing GOs concentration in all the GO types. Among them, DA‐GO and SDS‐rGO exhibited more pronounced toxicity compared to their parent compound at high concentrations above 100 μg/mL, whereas GO and rGO showed about 40% cell viability at the same concentrations. However, all types of GO showed minimal cytotoxicity at lower concentrations of 32 𝜇g/mL and below. There were no significant differences in cell viability according to exposure time (24 vs. 48 h).

In summary, GO, rGO, SDS‐GO, and SDS‐rGO did not show obvious cytotoxicity at low concentration, while GOs with functional groups revealed a severe, concentration‐dependent toxicity in the same range. High concentrations of all GOs resulted in severe toxicity to the A549 cells.

### 3.3 Autophagy‐Related Molecules and Activities as Detected by Western Blot in A549 Cells

**FIGURE 4:**
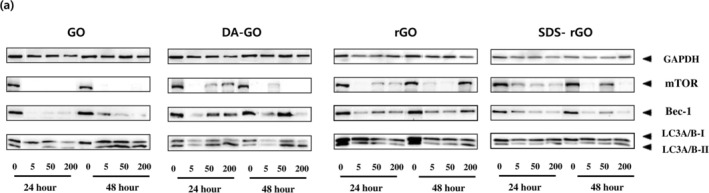
Western blot analysis results for each protein. Expression of GAPDH, mTOR, Beclin‐1, and autophagy marker proteins in A549 cells treated with different concentrations of graphene oxide derivatives for varying treatment periods.

**FIGURE 5:**
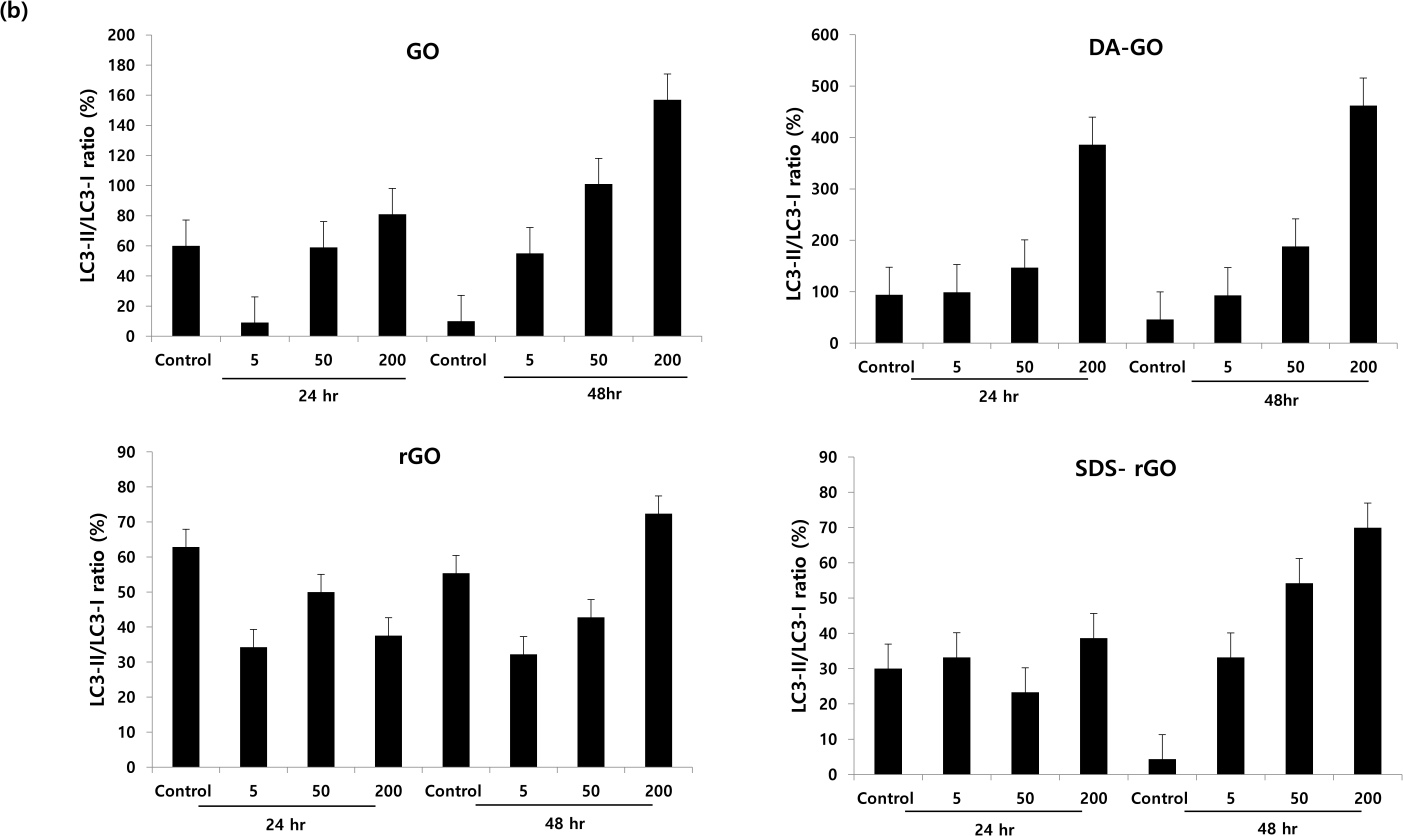
The conversion ratio of LC3I to LC3 II as a function of graphene oxide concentration and treatment duration.

Figure 4 demonstrates the western blot analysis results for each protein. Among many housekeeping genes, GAPDH served as an internal control for normalizing the semiquantitative analysis of protein expression because the expression of GAPDH level was constant in all the samples compared to expression of pan‐Actin level. Figure 5 shows the changes in the LC3A/B‐II to LC3A/B‐I ratio in cells treated with (a) GO, (b) DA‐GO, (c) rGO, and (d) SDS‐rGO. In autophagy activity, exposure of A549 cells to higher concentrations of four GO types increased the conversion ratio of LC3A/B‐I to LC3A/B‐II in a dose‐dependent manner. Exceptionally, this concentration‐dependent change of conversion was not observed in cells treated with rGO and SDS‐rGO for 24 h, while dose‐dependent trend was recovered in these cells for 48 h. The activity of autophagy in A549 cells tend to increase more at 48 h compared to 24 h.

**FIGURE 6:**
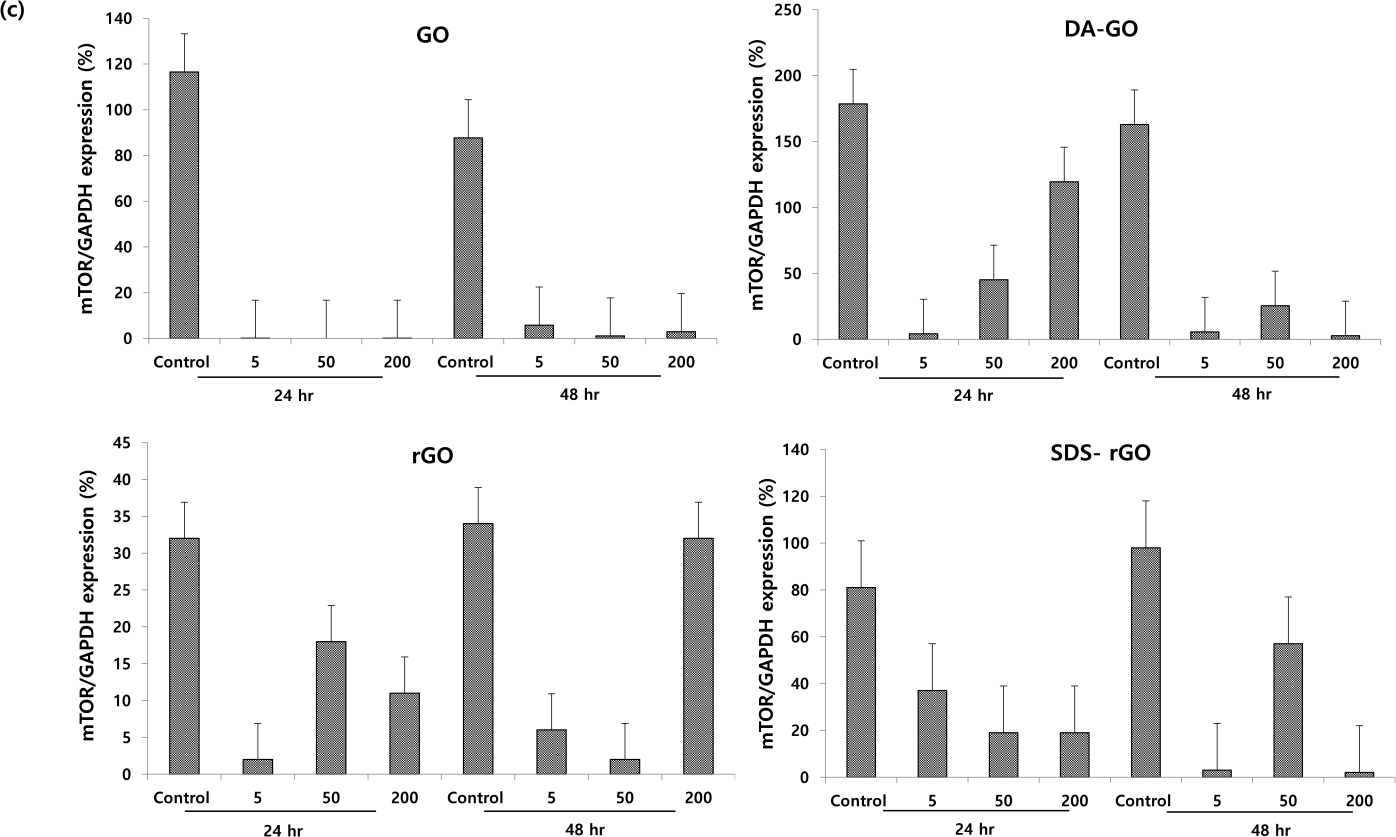
The expression levels of mTOR as a function of graphene oxide concentration and treatment duration.

Mammalian target of rapamycin (mTOR) is involved in the autophagy process, and it negatively regulates autophagy by inhibiting the induction step. As shown in Figure 6, the overall level of mTOR was decreased in A549 cells treated with all GOs compared to control, especially in GO. Also, the phosphorylated mTOR (p‐mTOR), activating form of mTOR, was rarely detected in all GOs.

**FIGURE 7:**
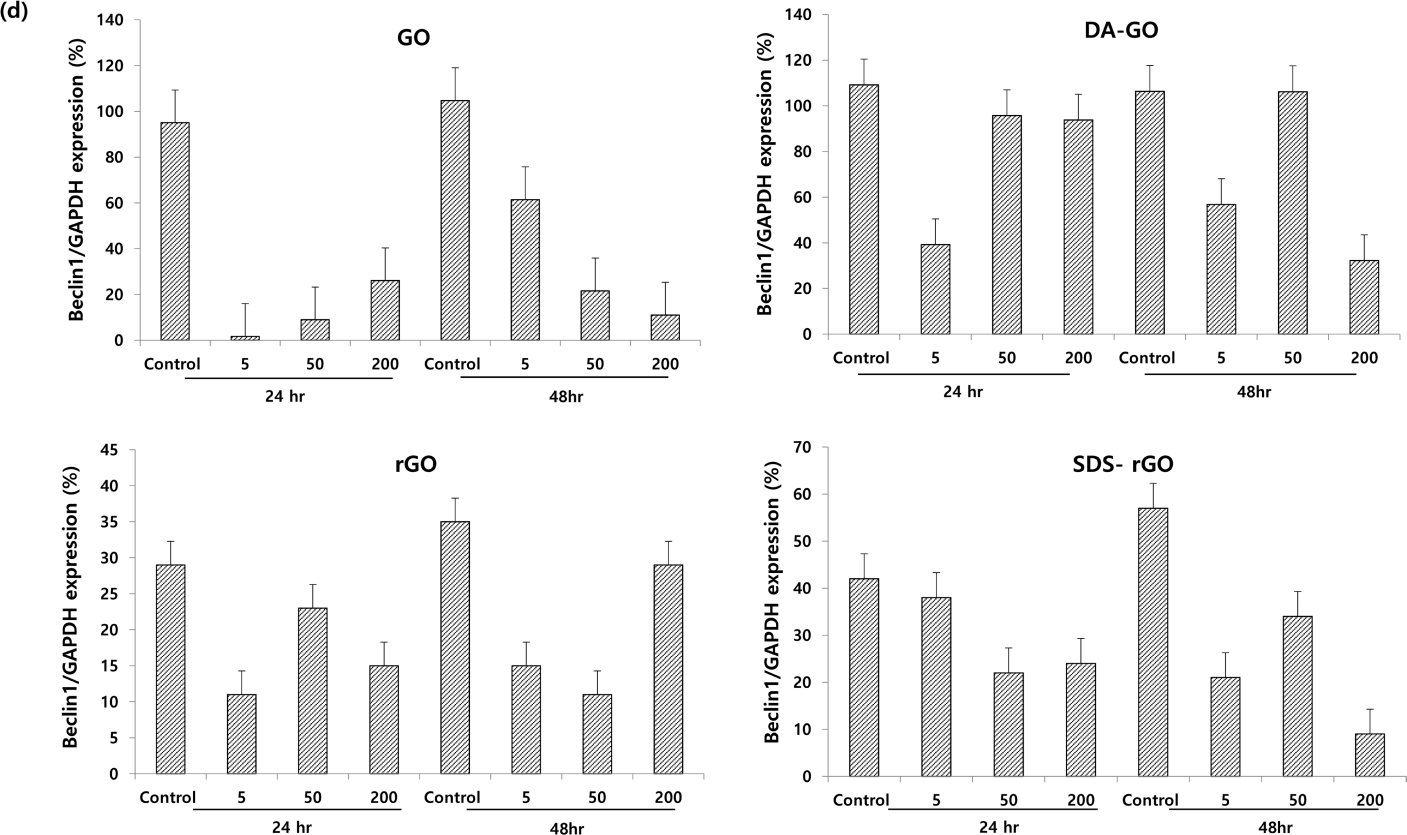
The expression levels of Beclin‐1 as a function of graphene oxide concentration and treatment duration.

Beclin‐1, a downstream effector of mTOR, was also decreased in A549 cells treated with all GOs compared to the control group. Both mTOR and Beclin‐1 expression levels decreased compared to the control regardless of concentration and exposure time, and they were not related to LC3A/B‐I to LC3A/B‐II expressions. Figure 7 also shows a bar graph of Beclin‐1 expression in A549 cells treated with all GOs.

## 4 Discussion

GO and its derivatives have distinctive physicochemical properties and can be used in various areas, including biomedical areas. Despite a wide range of applications in the biomedical field, their biological properties and activities in cells are still under study and have been poorly evaluated. In this study, we evaluated the toxicity and autophagy activity of various GO (GO, DA‐GO, rGO, and SDS‐rGO) exposure on A549 human lung carcinoma epithelial cells.

Cytotoxicity assessment of GO derivatives is essential to identify a safe dose range to avoid damage to normal cells, determine the appropriate dosage concentration and surface functionalization to maximize therapeutic effect, and use this data to establish a predictable safety profile in animal and clinical studies.

Firstly, we performed FTIR spectroscopy, which not only confirms the structure of the graphene derivatives but also allows us to predict their potential cytotoxicity and uptake mechanisms. GO shows strong –OH (≈3200–3600 cm^−1^), –COOH (≈1220–1260 cm^−1^), and epoxy (≈1000–1120 cm^−1^) peaks, indicating abundant oxygen‐containing groups. As a result, GO is highly hydrophilic and negatively charged at physiological pH, which both limits nonspecific binding to the A549 membrane and predicts relatively low cytotoxicity. In contrast, rGO displays markedly decreased O–H, C=O, and C–O–C bands due to reduction, reflecting loss of oxygen functionality. This makes rGO more hydrophobic and prone to aggregation, so that aggregated nanoplates can physically disrupt or porate the A549 membrane, which enhances ROS generation and cytotoxicity. DA‐GO exhibits a 2955‐,·2922‐,·and 2852‐cm^−1^ pattern from the long dodecyl‐amine hydrocarbon chain, which inserts into the lipid bilayer and induces membrane stress (promoting rapid clathrin‐mediated uptake). The 1644‐,·1457‐,·and 1265‐cm^−1^ peaks arise from amide‐bound amines; at physiological pH, these amines become –NH_3_^+^, strengthening electrostatic binding to the negatively charged membrane, triggering proton‐sponge–mediated endosomal rupture, ROS production, and it may induce higher cytotoxicity. The 1644‐, 1457‐, and 1265‐cm^−1^ peaks arise from amide‐bound amines; at physiological pH, these amines become –NH_3_^+^, strengthening electrostatic binding to the negatively charged membrane, triggering proton‐sponge–mediated endosomal rupture and ROS production, which may induce higher cytotoxicity. Finally, SDS‐rGO retains the rGO backbone (with diminished oxygen peaks) and shows an 1168 cm^−1^ S=O peak from residual SDS; this amphiphilic structure (hydrophilic sulfate head + hydrophobic tail) promotes membrane insertion, caveolae‐ or fusion‐like uptake (Chen et al. 2021), and enhanced endosomal fusion—further boosting ROS generation and making SDS‐rGO even more cytotoxic than rGO. In fact, our FTIR‐predicted trends matched MTT assay results, demonstrating that FTIR can serve as a useful supplementary test to forecast both cellular uptake behavior and relative cytotoxicity of graphene derivatives. In fact, in our study, the expected results of FTIR and MTT assay were similar, and we believe that FTIR may be used as a supplementary test to predict the cytotoxicity of graphene compounds.

Our results also demonstrated that GO and its derivatives had an effect on the survival of A549 cells depending on concentration through the MTT assay. These findings are consistent with those of previous studies (22, 28, and 29). In general, the cytotoxicity was reported at the GO concentration above 100–200 μg/mL. Chang et al. reported that GO did not induce obvious cytotoxicity, but the cell viability was decreased at 200 μg/mL of GO (Chang et al. 2011). In another study, exposure to 100 μg/mL of GO induced cell toxicity (Nasirzadeh et al. 2019; Schinwald et al. 2014; Zhang et al. 2012), and rGO treated cells also showed cell toxicity at the 100‐μg/mL concentration (Akhavan et al. 2012; Jaworski et al. 2015). In our study, all GOs were generally safe on A549 cells within physiologically accepted low concentrations (< 32𝜇g/mL), and only showed toxicity at high concentrations (> 100𝜇g/mL). In conclusion, when summarizing the previous studies and our study, low concentrations of GOs are safe in A549 cells.

Notably, DA‐GO and SDS‐rGO showed relatively more severe toxicity than the original form at high concentrations (> 100𝜇g/mL). These results were probably caused by its functional group. As mentioned above, GO has a –COO‐(carboxyl group) negative charge at pH 7.4, which repels the A549 cell membrane, resulting in slow uptake and predominantly trapped in endosomes/lysosomes, resulting in less ROS generation and therefore less membrane damage. This is thought to result in high cell viability of 80%–90% or more below 50 μg/mL and maintaining around 60% viability in the 50–100‐μg/mL range. On the other hand, as for DA‐GO, dodecylamine is hydrophobic cationic surfactants, and one study reported that this attached amine group (NH_2_) can cause DNA damage and cell toxicity (Chatterjee et al. 2016). This is probably due to the following mechanisms. When the amine group is positively charged (‐NH_3_^+^) at physiological pH, it binds strongly to the negatively charged cell membrane and is rapidly taken up, and at higher doses, membrane disruption and endosomal swelling occur, causing GO to leak into the cell and generate ROS in mitochondria and elsewhere. This results in only a minor reduction in survival at low concentrations, but at moderate and higher concentrations, the combination of membrane damage‐lysosomal leakage‐oxidative stress is thought to result in cell death of 50%–70% or more. Another functional residue, sodium dodecyl sulfate is an anionic surfactant, and it can also cause toxicity of human airway epithelial cell in vitro (Welch et al. 2021). The SDS‐coated rGO is thought to facilitate insertion and uptake into cell membranes due to its hydrophilic sulfate head and hydrophobic tail, which, at high concentrations, leads to a sharp drop in viability due to a combination of membrane disruption and ROS generation as mentioned above. Our study suggests that the concentration and functionalization of the GOs are one of the key factors in controlling cellular cytotoxicity. It is possible to reduce the toxicity of GO and unravel its broad range of applications in the biomedical field by controlling these factors.

We further evaluated the autophagy activity on A549 cells after 24 and 48 h of incubation. In our study, we showed that the ratio of conversion of LC3A/B‐I to LC3A/B‐II increased in a dose‐dependent manner on all graphene derivatives, suggesting that the autophagy response can be triggered after graphene exposure. Although the effect of graphene on autophagy activity in cells is still an area of ongoing research, some studies reported that graphene can trigger autophagy in many different cell lines, and our findings are consistent with the results (Chen et al. 2015; Chen et al. 2014; Liu et al. 2016). This response also increased in time‐dependent pattern, showing a stronger response observed at 48 h compared to 24 h. However, cells treated with rGO and SDS‐rGO showed an increase in autophagy activity in a dose‐dependent pattern after 48 h of treatment, while this trend was not observed at 24 h. Because the uptake of graphene into cells is affected by the particle size and surface chemistry (Zhang et al. 2016), this result may suggest that graphene substances with different properties may affect autophagy activity.

The hydrophilic properties of GO compared to rGO may enable continuous cell uptake (Ou et al. 2016). On the other hand, rGO aggregates easily due to its hydrophobicity, and aggregation of rGO may impede efficient cell uptake. Instead, hydrophobic rGO may adhere to the cell surface rather than being internalized, interacting with the cell membrane (Chatterjee et al. 2014; Jarosz et al. 2016; Siqueira et al. 2022; Zhang et al. 2016). Initially, autophagy activity may not be as prominent compared to the control group, but later on, there is a possibility that autophagy activity may increase as a result of the interaction between the cell membrane and the aggregated rGO.

Autophagy process is controlled by multiple intracellular molecules, and we evaluated upstream molecules including mTOR and Beclin‐1. The mTOR acts as a main negative regulator of the autophagy process by inhibiting the protein complex ULK1‐Atg13‐FIP200, which is required for the initiation of autophagosome formation. A previous study reported that a decrease in mTOR levels activates autophagy (Kim and Guan 2015), and we also observed a decrease in mTOR levels compared to the control groups in all GO‐treated cells. This result is consistent with an increase in the activity of autophagy in GO‐treated cells, suggesting that graphene materials are autophagy inducers.

**FIGURE 8:**
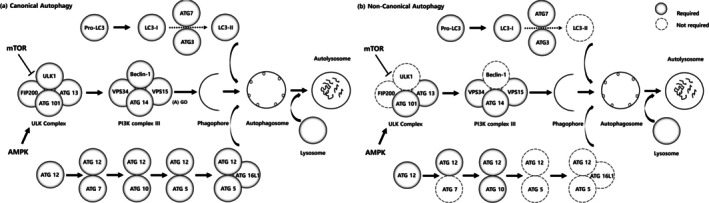
Schematic figure of the canonical pathway (a) and noncanonical pathway (b).

Beclin‐1 is another key protein that plays a critical role in the initiation of autophagosome formation. It is a positive regulator of autophagy, forming a complex with the class III PI3K (phosphatidylinositol 3‐kinase) VPS34 and other proteins. Typically, Beclin‐1 is a positive regulator of the autophagy process, and an increase in Beclin‐1 levels signifies an increase in autophagy activity (Kang et al. 2011). Interestingly, despite the increase in autophagy activity, Beclin‐1 level was actually suppressed compared to the control group in our study. Furthermore, the decrease in mTOR activity typically leads to phosphorylation of Beclin‐1, resulting in an increase in Beclin‐1 levels. However, in our study, Beclin‐1 level was also inhibited despite mTOR suppression. Although the relationship between these molecules is very complex and has not yet been fully studied, there are some possible explanations that could be considered. One possible explanation is that a noncanonical autophagy mechanism may be involved. Noncanonical autophagy pathways refer to the autophagic pathways that do not involve the traditional key regulator, such as Beclin‐1 and the Vps34 complex (Codogno et al. 2012). Figure 8 shows an illustration of the noncanonical autophagy pathway, which is different from the usual canonical autophagy pathway (Lindqvist et al. 2015). This noncanonical autophagy process can occur in response to stress situations such as reactive oxygen stress or DNA damage, and previous studies reported that autophagy can be activated without correlation to Beclin‐1. Previous studies reported that autophagy can be activated independently of Beclin‐1 when cells are exposed to preapoptotic compounds (Dupont and Codogno 2013). Autophagy induced by resveratrol has been shown to be positively linked to the death of a specific type of human tumor cell that does not rely on Beclin‐1 (Scarlatti et al. 2008), and the compound Z18, which specifically affects the BH3‐binding site of Bcl‐XL/Bcl‐2, has been demonstrated to activate autophagy in HeLa cells (Tian et al. 2010). Considering several studies that have reported that graphene exposure causes an increase in intracellular ROS generation and has a pro‐apoptotic effect (Krętowski et al. 2021; Shen et al. 2022; Sun et al. 2015), we speculate that graphene materials also promote autophagy activity in a Beclin‐1‐independent manner, like the aforementioned substances.

Another possibility is that the regulators of the apoptosis process affect autophagy activity. Beclin‐1 is regulated not only by mTOR but also by various substances, and it is known to cross‐link with the apoptosis process through substances such as Bcl‐2 (Marquez and Xu 2012). One theory is that the interaction between Beclin‐1 and Bcl‐2 exists. Under normal conditions, Bcl‐2 joins with Beclin‐1 to inhibit autophagy. However, under cell stress conditions, these two molecules dissociate, and Beclin‐1 binds to other autophagy‐related proteins to promote autophagy. Under cell stress conditions such as graphene exposure, oxidative stress is thought to occur and promote the dissociation of Beclin‐1 and Bcl‐2. Typically, this process induces the overexpression of Beclin‐1, but there can also be cases where the level of Beclin‐1 is actually suppressed or unchanged. According to a study by Chen et al., it has been shown that autophagy activity can be activated just by the separation of Beclin‐1 and Bcl‐2 through phosphorylation, even without an increase in Beclin‐1 expression level, like our study result (Chen et al. 2019).

**FIGURE 9:**
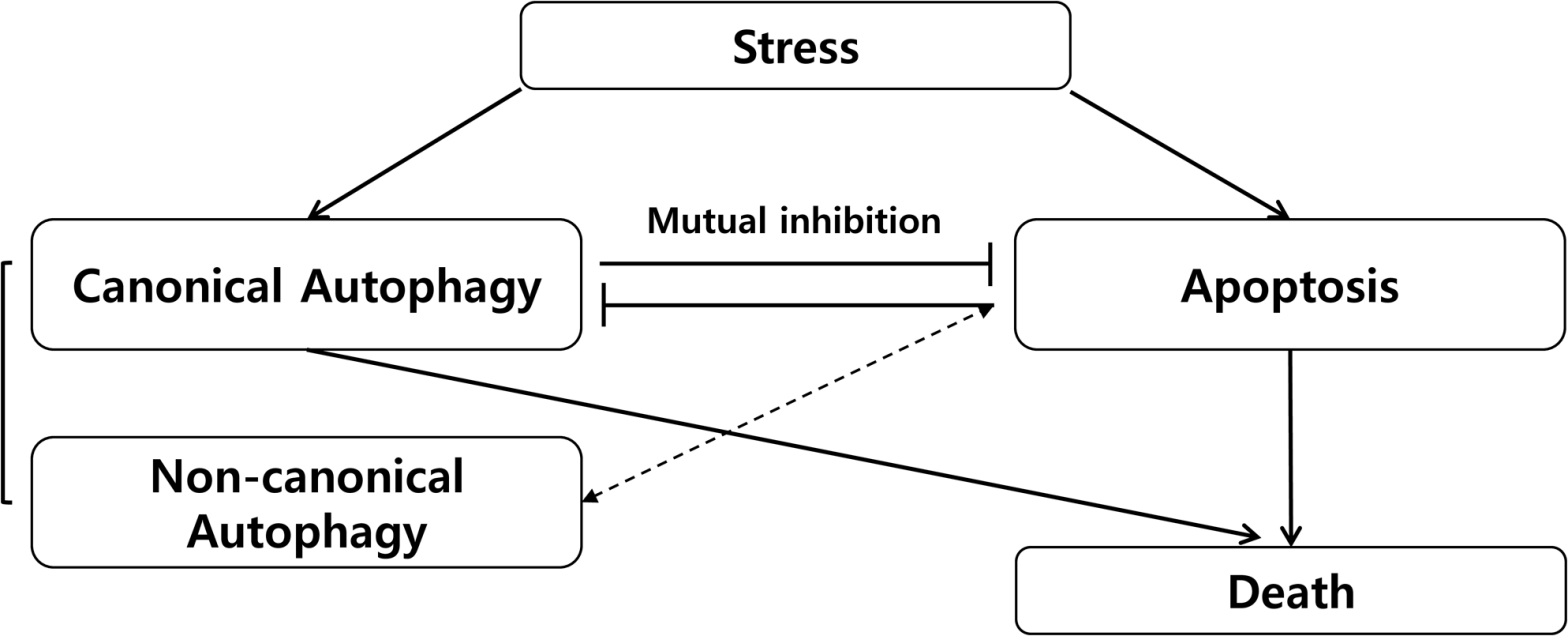
Possible mechanisms of autophagy regulation by graphene oxide derivatives.

Not only Bcl‐2, but also pro‐apoptotic proteins or activation of caspases released during apoptosis can result in the cleavage and degradation of Beclin‐1. Some studies have reported that caspase‐3 or caspase‐8, which occur during the apoptosis process, cleave Beclin‐1 (Li et al. 2011; Luo and Rubinsztein 2010; Zhu et al. 2010). Although cleavage of Beclin‐1 during apoptosis can inactivate autophagy, the autophagy activity can be increased through the cleavage of Atg4D by caspase‐3 (Kang et al. 2011). Although the relationship between autophagy and apoptosis is highly complex and dynamic, our result provides some insights into this relationship. In our study, we observed an increase in autophagy activity despite a decrease in Beclin‐1. Based on the fact that graphene materials can simultaneously induce both autophagy and apoptosis (Krętowski et al. 2021; Shen et al. 2022), it may be speculated that this apoptosis may inhibit the canonical autophagy pathway by suppressing Beclin‐1, but it may increase the noncanonical autophagy pathway through Beclin‐1 independent manners (Figure 9). But the exact and intricate mechanisms of the autophagy process remain unclear, and additional research is necessary to uncover them.

In summary, the toxicity of graphene derivatives is thought to occur by the following mechanisms. Firstly, the hydrophobic aggregates of rGO or SDS‐rGO and the long alkyl chains of DA‐GO induce insertion‐perforation into the lipid bilayer, compromising cell membrane integrity, and the amine groups of DA‐GO are converted to a positive charge (‐NH_3_^+^) at physiological pH, promoting clathrin‐mediated endocytosis (Ou et al. 2016). Osmotic swelling due to the proton sponge effect within the endosome then ruptures the endosomal membrane and releases the graphene sheet into the cytosol, where it interacts with mitochondria and reductases to excessively generate ROS. Excessive ROS oxidatively denature lipid‐protein‐DNA, initiating apoptosis (caspase‐8, 9/3 activation, and DNA fragmentation) while causing LC3‐II conversion (Ahamed et al. 2019, 2020a), mTOR inhibition and decrease in Beclin‐1, and inducing Beclin‐1‐independent autophagy to activate autophagy. These complex mechanisms are thought to result in different modes of toxicity depending on concentration and surface chemistry.

Overall, our findings investigated the cytotoxicity of graphene derivatives and their mechanisms in A549 lung cancer cells, which may help in approaches to improve their anticancer activity. Firstly, we can consider the usefulness of graphene derivatives as drug delivery carriers. Given the relatively high cell viability of GO, which is hydrophilic, negatively charged, and characterized by multiple carboxyl groups in FTIR, we believe that treating graphene with low‐toxicity coatings (e.g., carboxyl, PEG) for drug delivery could minimize nontarget cell damage (Ahamed et al. 2021). Second, as an intrinsic anticancer agent, GO incorporated with hydrophilic–hydrophobic complex structures such as amines (DA‐GO) or SDS can be harnessed for tumor killing as they induce rapid cellular uptake, endosome rupture, and excessive ROS generation. In fact, SnO_2_‐ZnO/rGO composites have shown more potent ROS‐mediated killing than ZnO alone in MCF‐7 breast cancer cells (Ahamed et al. 2021). By mapping the surface functional groups predicted by FTIR and the A549 cytotoxicity data, we believe that graphene derivatives can be rationally designed as materials with direct anticancer activity. Furthermore, our study showed that exposure of A549 cells to graphene compounds activated Beclin‐1‐independent autophagy in a concentration‐dependent manner, which may be relevant for anticancer drug development. Autophagy can act as a tumor‐suppressive mechanism through the removal of damaged organelles, but it also has the potential to protect cancer cells and induce anticancer drug resistance. Therefore, the development of anticancer drugs using graphene derivatives will require a strategy to carefully regulate autophagy to an appropriate level by modulating key factors in the Beclin‐1‐independent pathway to prevent autophagy from acting as a tumor cell protection mechanism as the concentration of graphene increases.

Our study has several limitations. First, A549 cells are a lung cancer cell line, which is not completely identical to real in vivo cells, so our results may not be generalizable to normal cells. Furthermore, the in vitro environment is different from the in vivo environment as it excludes factors such as blood flow and immune response and may not accurately reflect the actual toxicity mechanism. Finally, in vitro tests may produce inconsistent results if the experimental conditions, such as concentration, exposure time, and temperature, are different from those in vivo. In addition, the cell death mechanisms or cell–cell interactions observed in vitro do not fully account for the complex responses at the actual tissue level. Therefore, we believe that further studies are needed to accurately predict actual in vivo safety.

## 5 Conclusions

In conclusion, we investigated the cytotoxic effects and autophagy response of GO derivatives in A549 cells. The degree of graphene toxicity depended on concentration and functional group. The exposure to the graphene derivatives showed increased conversion of LC3A/B‐I to LC3A/B‐II, and the autophagy regulating proteins, including mTOR and beclin‐1, were downregulated. These findings suggest that graphene exposure may induce a Beclin‐1‐independent autophagy process through a noncanonical manner and apoptosis, and autophagy processes may interact and function together.

## Conflicts of Interest

The authors declare no conflicts of interest.

## Supporting information


**Table S1:** FTIR peaks summary for GO derivatives.

## Data Availability

The data that support the findings of this study.
