# Supplementary material for: Cell Toxicity and Autophagy in A549 Cells Treated With Surface‐Functionalized Graphene Derivatives
Source: J Appl Toxicol. 2025 Sep 9;46(3):821–32. doi: 10.1002/jat.4869 (PMC12861601; doi:10.1002/jat.4869)
Supplement: Supplementary file 1 — Table S1: FTIR peaks summary for GO derivatives. [file JAT-46-821-s001.docx]

**Supplementary Table 1: FTIR Peaks Summary for GO Derivatives**

| **GO Derivative** | **Indicator Peaks (Functional Groups)** | **O–H/C=O/C–O–C Intensity Trend** |
| --- | --- | --- |
| **GO** | O–H 3437; C=O 1700–1733; C–O–C 1272; C–O 1056 | Strong |
| **DA-GO** | C–H 2955/2922/2852; amide C=O 1644; N–H 1457/1265/3300–3500 | Moderate  –COOH/epoxy decrease |
| **rGO** | C–H out-of-plane 998/990; (O–H/C=O/C–O diminished) | Weak (◌) |
| **SDS-rGO** | S=O 1168; C–H out-of-plane 998/990; (O–H/C=O/C–O diminished) | Weak |
